# *Agaricus* Mushroom-Enriched Diets Modulate the Microbiota-Gut-Brain Axis and Reduce Brain Oxidative Stress in Mice

**DOI:** 10.3390/antiox11040695

**Published:** 2022-03-31

**Authors:** Josune García-Sanmartín, Miriam Bobadilla, Eduardo Mirpuri, Vanessa Grifoll, Margarita Pérez-Clavijo, Alfredo Martínez

**Affiliations:** 1 Oncology Area, Center for Biomedical Research of La Rioja (CIBIR), 26006 Logroño, Spain; jgarcias@riojasalud.es (J.G.-S.); mbobadilla@riojasalud.es (M.B.); emirpuri@riojasalud.es (E.M.); 2 Centro Tecnológico de Investigación del Champiñón de La Rioja (CTICH), 26560 Autol, Spain; microbiologia@ctich.com (V.G.); direccion@ctich.com (M.P.-C.)

**Keywords:** microbiota-gut-brain axis, neurodegenerative diseases, brain oxidative stress, *Agaricus*-supplemented diets, antioxidant activities, lipid peroxidation

## Abstract

Neurodegenerative diseases pose a major problem for developed countries, and stress has been identified as one of the main risk factors in the development of these disorders. Here, we have examined the protective properties against brain oxidative stress of two diets supplemented with 5% (*w*/*w*) of *Agaricus bisporus* (white button mushroom) or *Agaricus bisporus brunnescens* (Portobello mushroom) in mice. These diets did not modify the weight gain of the animals when compared to those fed with a regular diet, even after feeding on them for 15 weeks. The long-term modification of the microbiota after 12 weeks on the diets was investigated. At the phylum level, there was a large increase of *Verrucomicrobia* and a reduction of *Cyanobacteria* associated with the mushroom diets. No changes were observed in the *Firmicutes/Bacteroidetes* ratio, whose stability is a marker for a healthy diet. At the family level, three groups presented significant variations. These included *Akkermansiaceae* and *Tannerellaceae*, which significantly increased with both diets; and *Prevotellaceae*, which significantly decreased with both diets. These bacteria participate in the generation of microbiota-derived short-chain fatty acids (SCFAs) and provide a link between the microbiota and the brain. Mice subjected to restraint stress showed an upregulation of *Il-6*, *Nox-2*, and *Hmox-1* expression; a reduction in the enzymatic activities of catalase and superoxide dismutase; and an increase in lipid peroxidation in their brains. All these parameters were significantly prevented by feeding for 3 weeks on the *Agaricus*-supplemented diets. In summary, the supplementation of a healthy diet with *Agaricus* mushrooms may significantly contribute to prevent neurodegenerative diseases in the general population.

## 1. Introduction

The incidence of neurodegenerative diseases (NDs) is currently rising due to the increasing lifespan of the world’s population [1]. NDs encompass a heterogeneous group of chronic disorders that include Alzheimer’s disease (AD) and others dementias, such as Huntington’s disease, Parkinson’s disease [2], multiple sclerosis, human prion, and motoneuron diseases [3]. Following data from the World Health Organization, the number of people with dementia in 2015 was 46.8 million worldwide but is expected to rise to 131.5 million by 2050 [4]. Of these, AD is the most common ND, accounting for 60–70% of cases, which represents nearly 45 million people [5,6]. NDs are characterized by the progressive loss of vulnerable neurons in specific regions of the brain [7]. Heretofore, all these diseases represent the fourth cause of global disease burden in developed countries and lack an effective treatment [8].

AD is characterized by the loss of synapses and cortical neurons, which are due to the accumulation of amyloid-beta peptide and hyperphosphorylated Tau protein in the brain [9]. Aging, diabetes mellitus, and oxidative stress are known as the main risk factors of developing NDs [5,10], and oxidative stress is usually considered the more dangerous [11] due to the heavy dependence of the brain on a constant oxygen supply [12]. In contrast, cognitive and physical activities and adherence to the Mediterranean diet are associated with a decreased risk of developing AD [13].

Nowadays, growing evidence supports an association between lifestyle habits and a delay in AD occurrence [14]. In that regard, a healthy diet containing some drinks, such as wine and other fruit juices, and prebiotic foods, such as mushrooms or vegetables, exhibits positive properties with antioxidative and anti-inflammatory attributes [15,16,17,18]. Today, a new paradigm is gaining great support, namely the connection among diet, changes in the gut microbiota, and modifications on brain physiopathology [19,20]. This is currently known as the microbiota-gut-brain axis [21,22,23].

It has been shown that edible mushrooms modulate the gut microbiota towards a healthier composition [24]. The industrial cultivation of mushrooms of the *Agaricus* genus, including *A. bisporus* (white button mushroom) and *A. bisporus brunnescens* (Portobello or cremini mushroom), is an important staple of the economy for the region of La Rioja in Northern Spain [25]. The beneficial effects of this mushroom genus have been known for long, especially for *A. blazei* [26]. In the case of the white button mushrooms, several studies have established that their consumption induces a healthier microbiota [27], a better immunological function [28], and a reduction of obesity and fatty liver development [29]. No references were found for the potential relationship of Portobello mushrooms with the microbiota or for the connection of either white button or Portobello mushrooms with the microbiota-gut-brain axis.

Therefore, the aim of this study was to investigate the impact of *Agaricus* mushroom-enriched diets on the microbiota-gut-brain axis. To accomplish this, we followed two specific goals: to establish the influence of such diets in long-term microbiota composition, and the impact of the diets on the brain’s oxidative status.

## 2. Materials and Methods

### 2.1. Mushroom-Enriched Diets

Mushrooms (white button and Portobello) were produced at the Centro Tecnológico de Investigación del Champiñón de La Rioja (CTICH), following local industry standard cultivation methods. Clean carpophores were cut into small pieces, freeze-dried, and ground into a dry powder that was sent to Research Diets (New Brunswick, NJ, USA). Pellets for three different diets were generated under the supervision of professional nutritionists: (i) a regular basic diet containing 15% fat (D11112201, Open Standard Diet, Research Diets), (ii) a basic diet supplemented with 5% (*w*/*w*) freeze-dried white button mushroom powder, and (iii) a basic diet supplemented with 5% (*w*/*w*) freeze-dried Portobello mushroom powder. Nutritional composition of the three diets is shown in Table 1.

### 2.2. Experimental Animals, Feces Collection, and DNA Extraction

All procedures involving animals were carried out in accordance with the European Communities Council Directive (2010/63/EU) and Spanish legislation (RD53/2013) on animal experiments and with approval from the ethical committee on animal welfare of our institution (Órgano Encargado del Bienestar Animal del Centro de Investigación Biomédica de La Rioja, OEBA-CIBIR, procedure number AMR14).

In order to investigate the long-term impact of the diets on mouse microbiota, six-week-old C57BL/6J male mice (*n* = 30) (Charles River) were randomly divided into 3 groups and fed with the diets for 15 weeks. Mice were housed under standard conditions at a temperature of 22 °C (±1 °C) and a 12-h light/dark cycle with free access to food and water. Males were selected to avoid interferences with the estrous cycle. Individual mouse weights and cage food consumption were recorded weekly. All animals were housed in the same room under specific pathogen-free (SPF) conditions and maintained by the same personnel in order to normalize their microbiota. After 12 weeks, fresh fecal contents were collected from each animal and weighed. The DNA was subsequently extracted from fecal microbiota using the DNeasy Blood & Tissue Kit (Qiagen, Venlo, The Netherlands). DNA purity and concentration were determined by a Nanodrop spectrophotometer (ND-1000; Thermo Fisher Scientific, Waltham, MA, USA).

### 2.3. Bacterial 16S rDNA Massive Sequencing and Sequence Postprocessing

Samples were amplified for the 16S rDNA hypervariable region V3–V4, as previously described [30], following the protocol 16S Metagenomic Sequencing Library Preparation (Illumina, INC, San Diego, CA, USA) using a MiSeq ultra-sequencer (Illumina) and a 3430-cycle paired-end read run. Internal controls included the gut and soil microbiome standards (MSA-1006 and MSA-3001, ATCC, Manassas, VA, USA). The quality of the initial reads was evaluated with FastQC v0.11.9 [31]. The bioinformatics analysis was carried out using the different applications of protocol Qiime2 [32], as described [33]. Specifically, we used version qiime2-2021.4, installed through a Conda environment in platform Ubuntu Linux 20.04.2 LTS. For taxonomic assignation, we used the SILVA database in its latest version, 132 [34].

Alpha- and beta-diversity phylogenetic indexes were calculated. From these data, bacterial groups with differential abundance among diets were identified (ANCOM test) [35], and principal component graphs were drawn.

Metagenomic sequencing raw data have been deposited at [36].

### 2.4. Restraint Stress Model

In order to study the effects of the diets on brain’s oxidative stress parameters, additional mice (*n* = 30) (Charles River) were used for this assay. Mice were randomly divided into six experimental groups (three diets, exposed or not to stress, *n* = 5 per group) and fed with the diets for 3 weeks. After that time, animals were subjected to an acute model of stress by immobilization, as previously described [17,18]. Briefly, mice were placed inside 50-mL conical tubes, with adequate ventilation and with no access to food or water for 6 h. Due to the corticosterone circadian rhythm, restraint stress was applied at the same time of the day (9:00 a.m.) in all experiments.

Upon finishing the stress, animals were immediately sacrificed. The whole brain was divided into two equal halves by a sagittal section. One side was used for RNA extraction and the other one for an antioxidant enzyme analysis (see below).

### 2.5. RNA Extraction and qRT-PCR

Total RNA was isolated from mouse brains and purified as described [17]. Briefly, total RNA was isolated using Trizol reagent (Invitrogen, Waltham, MA, USA), and the resulting RNA (5 µg) was reverse-transcribed using the Superscript III First-Strand Synthesis System for RT-PCR (Invitrogen). cDNA was amplified using SYBR Green PCR Master Mix (Applied Biosystems, Foster City, CA, USA) in a real-time PCR (7300 Real-Time PCR System, Applied Biosystems). For each transcript, a specific calibration curve was included to analyze the expression of *Nox-2*, *Hmox-1*, *Il-6*, *Tnfα*, and *Nrf2*. As a housekeeping gene, *Gapdh* was used. Specific primers are shown in Table 2.

### 2.6. TBARS, SOD, and Catalase Activity

For the determination of the oxidative stress parameters and antioxidant components in the brain, frozen tissues were homogenized (RIPA buffer, Thermo Scientific) supplemented with Complete and Phospho STOP (Roche, Basel, Switzerland) protease and phosphatase inhibitors), as described [18]. The activities of superoxide dismutase (SOD) and catalase were studied with commercially available kits (CA061 and CA063, respectively, Canvax, Córdoba, Spain). Lipid peroxidation was determined using a commercial TBARS assay kit (CA995, Canvax). The final products were detected with a microplate reader (POLARstar Omega, BMG Labtech, Ortenberg, Germany) at 530/590 nm (for catalase and TBARS) or 450 nm for SOD.

### 2.7. Statistical Analysis

For comparisons, the data distribution was checked for normality (Shapiro–Wilk test) and homoscedasticity (Levene test). When datasets were normally distributed with equal variances, one-way or two-way ANOVA was used. A nonparametric Kruskal-Wallis test was used for not-normal data. The significance level was set at *p* < 0.05.

## 3. Results

### 3.1. Mushroom-Enriched Diets Do Not Modify Mouse Body Weight in the Long Term

**Figure 1:**
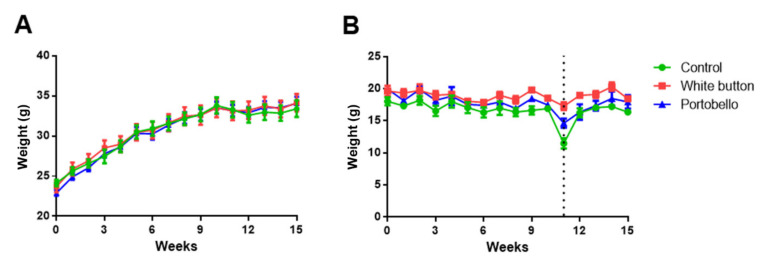
Weight gain (**A**) and food consumption (**B**) by all experimental animals throughout 15 weeks of study. At week 11 (dotted line), animals had to be transferred to larger cages, and a transient diminution in food consumption was observed. Each value represents the mean and standard deviation for all animals (*n* = 10 per group).

The weights of the animals fed with the three experimental diets were followed for 15 weeks. No significant differences were found in the weights of the animals subjected to the different diets (Figure 1A). Food consumption was constant during the whole period, with no significant differences among the diets (Figure 1B). At 11 weeks, mice had to be transferred to larger cages due to their increasing size. This change induced a transitory diminution in food consumption that was corrected by the following week (Figure 1B).

### 3.2. Mushroom-Enriched Diets Induced a More Beneficial Microbiota Composition

**Figure 2:**
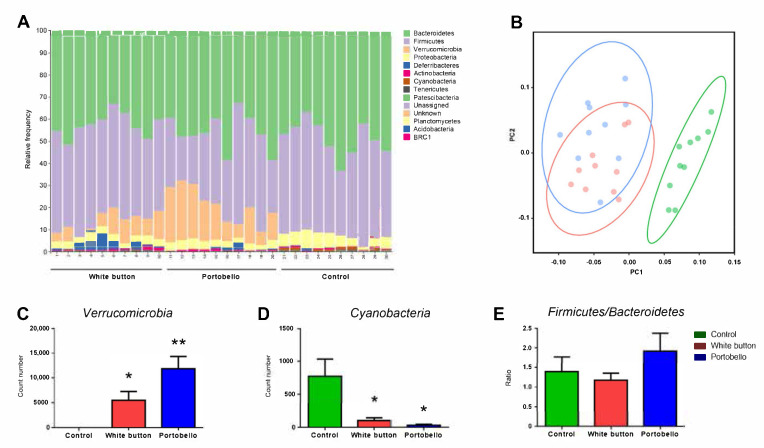
Relative frequency of bacteria at the phylum level. A general comparison of all phyla in the experimental animals fed with the white button diet (1–10), Portobello diet (11–20), or basic diet (21–30) shows a clear upregulation of *Verrucomicrobia* (orange) associated with the diets (**A**). A principal component analysis shows a marked difference between the control diet (green) and the other two diets (**B**). Quantification of *Verrucomicrobia* (**C**) and *Cyanobacteria* (**D**) shows also clear differences with the control diet. There were no significant differences in the *Firmicutes/Bacteroidetes* ratio (**E**). Bars represent the mean and standard error of the mean (SEM) of all measurements (*n* = 10). *: *p* < 0.05 and **: *p* < 0.01 versus control.

The microbiota of mice that were exposed to the experimental diets for 12 weeks were identified by massive sequencing and biostatistical analysis. At the phylum level, there was a large increase of *Verrucomicrobia* associated with the mushroom diets (*p* = 0.014; *p* = 0.001 for white button- and Portobello-enriched diets compared to the control, respectively), which was more important in mice that took the Portobello-enriched diet (Figure 2A,C). In addition, the frequency of the phylum *Cyanobacteria* significantly diminished with the diets (*p* = 0.033; *p* = 0.021, respectively) (Figure 2D). These differences translated into clearly separated data point groups in a principal component analysis for the supplemented diets vs. the basal one (Figure 2B). The *Firmicutes*/*Bacteroidetes* ratio has been identified as a potential clinical marker for dysbiosis [37], so it was also calculated (Figure 2E). No significant differences were found between the mushroom diets and the control (*p* = 0.584 and *p* = 0.391, respectively).

**Figure 3:**
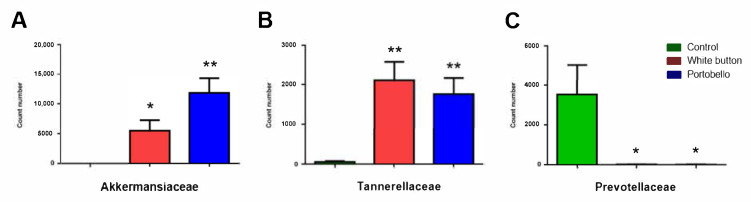
Count numbers for some bacterial families, including *Akkermansiaceae* (**A**), *Tannerellaceae* (**B**), and *Prevotellaceae* (**C**). Bars represent the mean and standard error of the mean (SEM) of all measurements (*n* = 10). *: *p* < 0.05 and **: *p* < 0.01 versus control.

At the family level, three groups presented significant variations. These included *Akkermansiaceae* (Figure 3A), *Tannerellaceae* (Figure 3B), and *Prevotellaceae* (Figure 3C). The *Akkermansiaceae* family belongs to the *Verrucomicrobia* phylum. In parallel with the phylum data, the counts of *Akkermansiaceae* in basic diet-fed mice were very low but became significantly higher in animals that were fed with the white button diet (*p* = 0.014) or with the Portobello diet (*p* = 0.001) (Figure 3A). The pattern of the *Tannerellaceae* family (phylum *Bacteroidetes*) frequency was similar to that of *Akkermansiaceae*, with low counts for the basic diet and significant increases for both mushroom-enriched diets (*p* = 0.002 and *p* = 0.003, respectively) (Figure 3B). On the contrary, the *Prevotellaceae* family (also from the *Bacteroidetes* phylum) displayed a significant count reduction associated with the mushroom diets (*p* = 0.043 for both) (Figure 3C).

### 3.3. Mushroom-Enriched Diets Reduced the Expression of Stress-Related Genes

**Figure 4:**
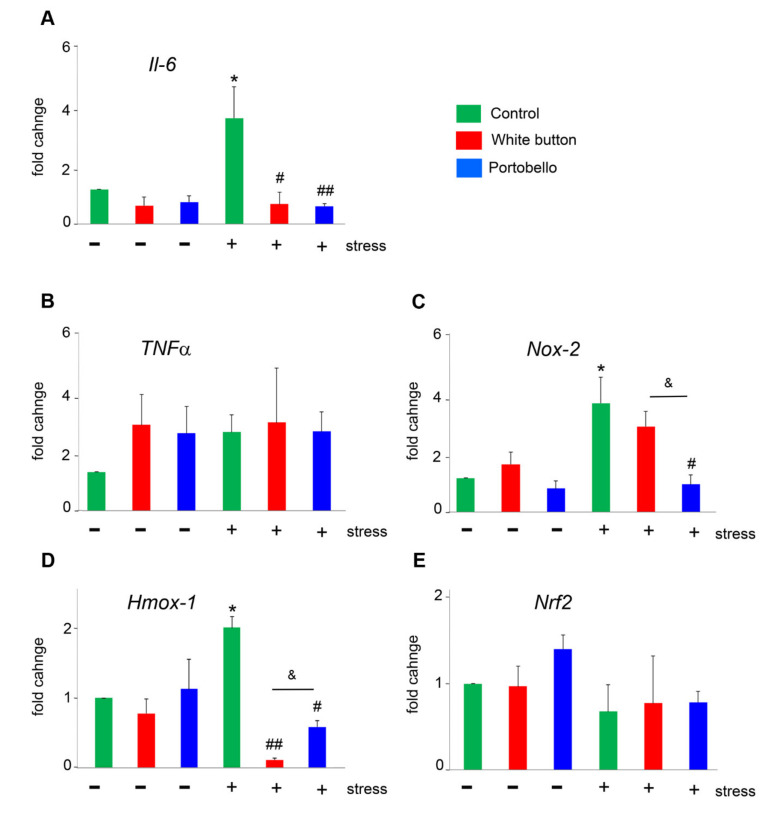
Expression of genes related to oxidative stress, including *Il-6* (**A**), *Tnfα* (**B**), *Nox-2* (**C**), *Hmox-1* (**D**), and *Nrf2* (**E**). Bars represent the mean and standard error of the mean (SEM) of all measurements (*n* = 5). *: *p* < 0.05 versus control. #: *p* < 0.05 and ##: *p* < 0.01 versus the stressed control. &: *p* < 0.05 between diets.

The brains of mice that were exposed to the diets for 3 weeks were analyzed by qRT-PCR for several genes related to stress management (Figure 4). The expression of *Il-6*, *Nox-2*, and *Hmox-1* was significantly upregulated by stress (*p* = 0.036, *p* = 0.036, and *p* = 0.014, respectively), and that elevation was partially or totally prevented by the mushroom diets. When measuring *Il-6* expression, both mushroom diets intensely reduced stress-mediated *Il-6* overexpression (*p* = 0.016 for white button and *p* = 0.001 for Portobello) (Figure 4A). In the case of *Nox-2*, the Portobello diet reduced the expression to values similar to the unstressed controls (*p* = 0.037 versus the stressed control), but the white button diet did not have any significant effect (Figure 4C). For *Hmox-1*, both diets reduced its overexpression, but the white button diet was significantly more effective (*p* = 0.001) than the Portobello one (*p* = 0.027) (Figure 4D). The diets had no significant effects on the expression of *Tnfα* or *Nrf2* (Figure 4B,E).

### 3.4. Mushroom-Enriched Diets Normalized Stress-Induced Changes in Enzyme Activities and Lipid Peroxidation

**Figure 5:**
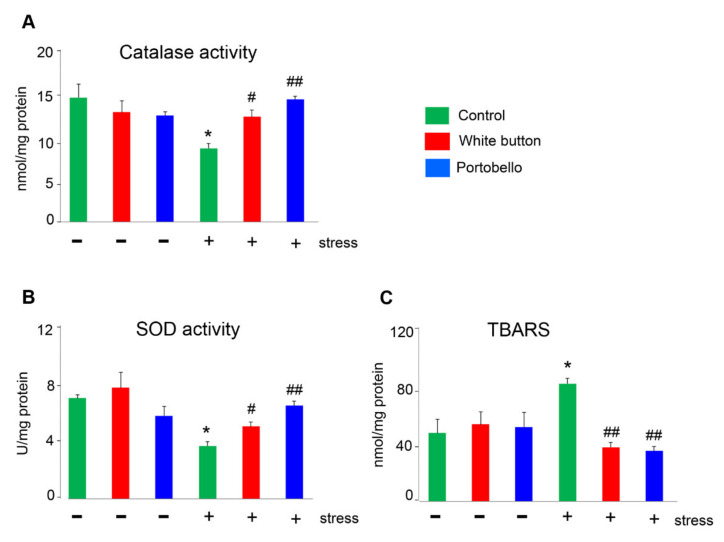
Quantification of antioxidant enzymes catalase (**A**) and SOD (**B**), and lipid peroxidation (**C**) in the brains of experimental mice. Bars represent the mean and standard error of the mean (SEM) of all measurements (*n* = 5). *: *p* < 0.05 versus the control. #: *p* < 0.05 and ##: *p* < 0.01 versus the stressed control.

Catalase and superoxide dismutase (SOD) activities were significantly reduced by stress (*p* = 0.036, *p* = 0.027, respectively) (Figure 5A,B). On the other hand, lipid peroxidation (TBARS), measured as the MDA levels, was significantly elevated by stress (*p* = 0.042) (Figure 5C). Both mushroom diets were able to elevate catalase and SOD enzymatic activities (Figure 5A,B) and to reduce lipid peroxidation (Figure 5C) to levels comparable with the unstressed brain. In the absence of stress, neither diet had a significant effect on these parameters (Figure 5).

## 4. Discussion

In this study, we have found that mushroom-enriched diets containing two variants of the *Agaricus bisporus* species exert a positive effect on microbiota composition, and they strongly contribute to reduce oxidative stress in the brain. These results suggest that supplementing a healthy diet with *Agaricus* mushrooms may be a beneficial habit for the general population.

We used metagenomics to understand the evolution of the microbiota in the gut of mice subjected to the diets, but a combination of different omics (metatranscriptomics, metaproteomics, and metabolomics, in addition to metagenomics) would provide a more detailed description of microbiota–host interactions [38]. This multiomics approach may strengthen our results and validate what pathways link microbiota modulation, oxidative stress, mitochondria physiology, and neurodegeneration.

Mushrooms, as well as natural products and medicinal plants, have important effects on the gut microbiota composition and, through it, modulate inflammation, cancer, and other diseases [39,40]. The modulation of the microbiota seems to be due to the prebiotic potential of mushroom components, including polysaccharides, terpenoids, and other bioactive compounds [41,42]. Our present data indicate that *Agaricus* mushrooms also have a positive influence in the composition of the microbiota, and through it, they contribute to reducing brain oxidative stress.

We also showed that supplementing a healthy diet with up to 5% of *Agaricus* mushrooms does not have an effect on mouse weight, even after 15 weeks. A previous study showed that *A. bisporus* can reduce obesity and accumulation of fat in the liver when experimental animals are exposed to a high-fat diet [29]. Therefore, the combination of a healthy diet and the intake of *Agaricus* mushrooms seems to be a good habit.

We demonstrated that the *Agaricus*-supplemented diets were able to modulate the microbiota towards a healthier composition. Although the study of microbiota variations and their association with different diseases is still in its infancy, several parameters are rising as potential clinical markers, including the *Firmicutes/Bacteroidetes* ratio [37]. This ratio seems to be critical to maintaining a healthy intestinal homeostasis, and both its increase and its reduction are regarded as dysbiosis, with the former associated with obesity and the latter with inflammatory bowel disease and cancer [43,44]. Interestingly, exposure to a mushroom-enriched diet did not modify this parameter, thus indicating that such diets are healthy for the consumers.

In addition, we found a very significant increase of the *Verrucomicrobia* phylum in those animals that consumed the mushroom diets. This phylum is considered a marker for a healthy microbiota, and the reduction of its frequency is related to dysbiosis, including children with irritable bowel syndrome [45]. Therefore, the increase we found after feeding the mice with the mushroom-enriched diets suggests a positive effect. We also found a significant reduction in the frequency of phylum *Cyanobacteria* associated with the diets. This phylum has been found to increase in patients with colon adenomas [46], so their reduction with the *Agaricus*-enriched diets seems to be a positive finding. Furthermore, some *Cyanobacteria* found in the environment may produce cyanotoxins, which are highly neurotoxic [47]. Whether some *Cyanobacteria* from the gut microbiota can generate such toxins is unknown.

When studying the frequency of different families of bacteria, three families showed a significant modulation by the diets. These included *Akkermansiaceae*, *Tannerellaceae*, and *Prevotellaceae*. Members of the *Akkermansiaceae* family are considered promising probiotics [48]; there is an inverse correlation between *Akkermansia* and obesity [49], and decreased metabolic parameters (insulin resistance, insulinemia, and cholesterol) were associated with this bacterial family in a clinical trial [50]. The positive effects of *Akkermansia* are due to several reasons, including the stimulation of mucosal microbial networks and the improvement of the intestinal barrier function, providing crucial host immunological responses. Several studies have demonstrated the possible involvement of *Akkermansia* reduction in the development of intestinal and metabolic disorders. Indeed, its levels inversely correlate to inflammatory bowel disease, obesity, and diabetes. Conversely, the therapeutic administration of *Akkermansia* decreases the development of such diseases [51,52].

The *Tannerellaceae* family includes anaerobic, Gram-negative bacterial species that have been implicated in periodontal diseases and are associated with esophageal cancer [53]. On the other hand, the presence of this group of bacteria in the gut seems to have a beneficial effect. For instance, in a mouse model of collagen-induced arthritis, *Tannerellaceae* were less abundant in the diseased animals than in the healthy ones [54]. In addition, *Tannerellaceae* species seem to be influenced by the amount of gluten in the food. For instance, *Tannerellaceae* increased in the microbiome of mice fed a gluten-containing diet, whereas *Akkermansiaceae* species increased in the intestinal microbiomes of mice fed a gluten-free diet [55]. In summary, the increase in the frequency of the *Tannerellaceae* family found in our mushroom-fed mice seems to be a positive trait for their microbiota composition.

Changes in the levels of bacteria belonging to the *Prevotellaceae* family can have different consequences. For instance, the levels of this bacterial group decrease in colorectal cancer models [56,57] but increase in children with irritable bowel syndrome [45], indicating that other parameters related to the specific disease may influence the effects of the bacteria. In our case, the mushroom diets induced a clear decrease in the members of this bacterial family. Given the positive effect of the diets on the overall brain stress markers, we could infer that the decrease in the number of *Prevotellaceae* bacteria may be a positive event for the modulation of the microbiota-gut-brain axis.

Oxidative stress is recognized as a very significant contributor to the pathogenesis of many devastating NDs [58]. In particular, mitochondrial dysfunction leads to the aberrant production of reactive oxygen species (ROS), which are capable of oxidizing lipids and proteins, ultimately causing cell death [59]. To model this problem in rodents, acute restraint stress has been commonly used, since it stimulates several cellular events, resulting in enhanced ROS production in the brain [60]. It has been shown that the extracellular release of ROS triggers inflammatory processes [61] that, finally, enhance the local production of cytokines IL-1β, IL-6, and TNFα [62]. NOX-2 is known for generating superoxide molecules under oxidative stress-mediated circumstances, and HMOX-1 acts as an oxidative stress-induced heat shock protein [63]. In our results, the expression of both genes (*Nox-2* and *Hmox-1*) increased in the brains of stressed mice, which agrees with previous reports [17,18,64]. The fact that the *Agaricus*-supplemented diets brought these levels to normal clearly indicates the powerful antioxidant activity of these supplements.

Based on our previous experience [17,18], we were expecting an increase in *Tnfα* and a reduction in *Nrf2* levels as a response to restraint stress. Although there were trends in that direction, no significant differences were observed in this study. Perhaps the stress was not strong or long enough to fully modulate these markers.

Furthermore, restraint stress precipitates many neurochemical, hormonal, and behavioral abnormalities that are associated with an imbalance of the brain’s intracellular redox state. Many authors have shown that restraint stress enhances lipid peroxidation and decreases the antioxidant enzyme activities in rodent brains [17,60]. SOD and catalase are the best-known antioxidant enzymes in the brain [65], and we found that the *Agaricus*-supplemented diets significantly increased the stress-reduced enzymatic activity of both proteins to levels undistinguishable from the non-stressed animals. On the other hand, lipid peroxidation is one of the main consequences of excessive brain ROS levels. MDA is one of the end products of such an activity and is commonly used as a marker of oxidative stress [66]. In this line, we found a significant increase of MDA in the brains of stressed mice that was prevented by the diets. All these data demonstrate the strong protective effect of the *Agaricus*-supplemented diets on brain stress and point to their beneficial effect on the diet.

The beneficial effects of the gut bacteria on the brain seem to be mediated, at least partially, by the microbiota-derived short-chain fatty acids (SCFAs). These are monocarboxylic acids with 2–5 carbons that are the products of the anaerobic fermentation of indigestible polysaccharides, such as dietary fiber and resistant starch [67]. Acetate, propionate, and butyrate are the main SCFAs created in the human gut [68]. After their formation, SCFAs are taken by colonocytes through monocarboxylate transporters [69]. These SCFAs are not metabolized in the colonocytes but are transmitted through the portal circulation into the liver, where they are used as an energy substrate for hepatocytes [70]. However, an important fraction of the SCFAs reaches systemic circulation and the brain. *Akkermansia* and *Prevotella* have been shown to participate in the generation of propionate and butyrate [71].

α-L-rhamnosidases are enzymes able to release aglycones and glucose by cleaving the terminal α-L-rhamnoses present in glycosylated phenolic molecules [72]. Since the human intestine possesses no rhamnosidase activity [73], the vast majority of these rhamnose glycosides remains unabsorbed and reaches the colon. The ability to transform specific rhamnose glycosides, such as hesperidin and rutin, has been demonstrated in some probiotic bacteria, including *Tannerellaceae* [74]. In the human body, L-rhamnose is converted into propionate, which has anti-inflammatory and antioxidant effects [75]. Of course, an excessive production of *Tannerella*-induced propionate, such as that generated during gingival infections, may induce NDs such as AD [76].

The interactions among the microbiota, the bowel, and the brain are complex. The bacteria can send biochemical signals directly to the brain (SCFAs) or to the gut (Toll-like receptors), which will communicate with the brain via spinal and vagal visceral afferent pathways. On the other hand, the brain would send sympathetic and parasympathetic inputs to the gut and, from there, influence the microbiota through the motility and secretion of bacteria-controlling peptides. However, nowadays, given the very complex relationships, there seems to be a tripartite conversation among the three components rather than a simple bidirectional process [22].

## 5. Conclusions

In summary, diets supplemented with either white button or Portobello mushrooms showed great potential in reducing oxidative stress markers in the brain, and they seem to do this through a modulation of the microbiota towards a healthier composition. The combination of physical activity [77] and a healthy diet supplemented with *Agaricus* mushrooms may significantly contribute to the cognitive health of the general population.

## Figures and Tables

**Table 1:** Nutritional composition of the three diets used in this study.

	Basic Diet (D11112201)		White Button Suppl.		Portobello Suppl.	
	grams (%)	kcal (%)	grams (%)	kcal (%)	grams (%)	kcal (%)
Protein	19	20	18	20	18	20
Carbohydrate	63	65	60	65	60	65
Fat	7	15	6	15	6	15
Total		100		100		100
kcal/gram	3.81		3.62		3.62	
Ingredients	grams	kcal	grams	kcal	grams	kcal
Casein	200	800	200	800	200	800
L-cystine	3	12	3	12	3	12
Corn starch	381	1524	381	1524	381	1524
Maltodextrin 10	110	440	110	440	110	440
Dextrose	150	600	150	600	150	600
Cellulose, BW200	75	0	75	0	75	0
Inulin	25	37.5	25	37.5	25	37.5
Soybean oil	70	630	70	630	70	630
Mineral mix S10028 (*w*/*o* Ca, P, or K)	10	0	10	0	10	0
CaKPO_4_	13	0	13	0	13	0
CaCO_3_, Light, USP	5.5	0	5.5	0	5.5	0
Potassium citrate	16.5	0	16.5	0	16.5	0
Vitamin mix V10001	10	40	10	40	10	40
Choline bitartrate	2	0	2	0	2	0
White button powder	0		56.5		0	
Portobello powder	0		0		56.5	
Total	1071.05	4084	1127.55	4084	1127.55	4084

**Table 2:** Primers used in this study. Annealing temperature was 60 °C for all transcripts.

Gene	Forward Primer (5′–3′)	Reverse Primer (5′–3′)	Accession No.
*Nox-2*	GCTGGGATCACAGGAATTGT	CTTCCAAACTCTCCGCAGTC	NM_007807
*Hmox-1*	TGCTCGAATGAACACTCTGG	TAGCAGGCCTCTGACGAAGT	NM_010442
*Il-6*	ATGGATGCTACCAAACTGGAT	TGAAGGACTCTGGCTTTGTCT	NM_031168
*Tnf* *α*	CCACCACGCTCTTCTGTCTA	CACTTGGTGGTTTGCTACGA	NM_001278601
*Nrf2*	AGCGAGCAGGCTATCTCCTA	TCTTGCCTCCAAAGGATGTC	NM_010902
*Gapdh*	CATGGCCTTCCGTGTTCCTA	GCGGCACGTCAGATCCA	NM_008084

## Data Availability

Metagenomic sequencing raw data was deposited at http://www.ncbi.nlm.nih.gov/bioproject/814431 (accessed on 1 March 2022).
